# Putative source and niche shift pattern of a new alien ant species (*Odontomachus troglodytes*) in Taiwan

**DOI:** 10.7717/peerj.14718

**Published:** 2023-02-06

**Authors:** Tzong-Han Lin, Kai-Wei Chan, Feng-Chuan Hsu, Chung-Chi Lin, Hui-Yun Tseng

**Affiliations:** 1 School of Life Science, National Taiwan Normal University, Taipei, Taiwan; 2 Department of Entomology, National Taiwan University, Taipei, Taiwan; 3 Institute of Ecology and Evolutionary Biology, National Taiwan University, Taipei, Taiwan; 4 Department of Biology, National Changhua University of Education, Changhua, Taiwan

**Keywords:** Environmental niche models, Exotic species, Niche expansion, Phylogenetic relationship, Trap-jaw ant

## Abstract

Alien species may pose substantial impacts on biodiversity around the globe through international trade and travel. A niche shift hypothesis was proposed to explain the adaptive change of alien or invasive species in new habitats. However, whether niche shifts occur in alien species likely depends on both characteristics inherent to the species itself and its original distribution. Here we identified a newly exotic trap-jaw ant (*Odontomachus troglodytes*) in Taiwan by morphological and phylogenetic analyses. The possible distribution range and the niche shift pattern were evaluated using ecological niche modelling. The results indicated that exotic *O. troglodytes* in the newly distributed area displayed a significant niche shift with low niche overlap and high niche expansion. This study reveals a long-distance invasive event from central Africa to Southeast Asia (more than 10,000 km) and predicts the potential distribution range of this new alien species in Taiwan.

## Introduction

Rapid long-distance travel and international trade have reshaped biodiversity worldwide in the Anthropocene. Species across several taxa have been shown to have the ability to access new habitats through human transportation networks, resulting in a breakdown of biogeographic barriers and an increasing number of invasive species over the last decades (Hulme, 2009; Capinha et al., 2015; Seebens et al., 2017). Among different taxa, the spread and invasion of ants (Hymenoptera: Formicidae) are strongly correlated with human activities. Globalization of some ants may be attributed to their small size, diverse ecological niche, eusocial characteristic, and polygynous mating system (Bertelsmeier et al., 2017; Abril & Gómez, 2020; Bertelsmeier, 2021).

In invasion ecology, the niche conservatism hypothesis suggests alien species retain ancestral ecological niches and occupy new regions with environmental factors similar to their native range (Wiens & Graham, 2005; Liu et al., 2020). In contrast, the niche shift hypothesis is applied to explain the greater tolerance and adaptation to new environments of invasive species during range expansion (Broennimann et al., 2007; Da Mata et al., 2010; Tingley et al., 2014). In addition, a recent study found that niche size and native range affect niche shift, with species with smaller native range sizes experiencing the most significant niche shift (Bates, Ollier & Bertelsmeier, 2020).

The ant genus *Odontomachus* (Hymenoptera: Formicidae: Ponerinae) comprises more than 70 species and is characterized by their “trap-jaw”, referring to their long, spring-loaded mandible (Larabee et al., 2016; Fernandes et al., 2021). Large body size and highly specialized mandible that can open at 180 degrees make them easily recognized in the wild. In Taiwan, *O. monticola* (*O. rixosus* group) is the only known species of the genus (Terayama, 2009), commonly seen on low to mid-altitude forest floors. In 2017, amateurs collected a species belonging to the *O. haematodus* group in southwestern Taiwan. After that, the ant became much more common and may have succeeded in colonizing Taiwan as many additional nests were discovered within a 24 km^2^ area (K-W Chan, pers. obs., 2021). The ant is characterized by their stout mandible with short, blunt subapical teeth, strongly striated body sculpture and 4, 3 palp formula (Brown Jr, 1976; Sorger & Zettel, 2011). The discovery of a conspicuous species close to the port and suburban habitats indicated that the species was likely introduced by humans.

Previous studies have found that several members of the *O. haematodus* group have established populations outside of their native range (Brown Jr, 1976; Fisher & Smith, 2008; Framenau & Thomas, 2008; Herrera, Longino & Dekoninck, 2014; MacGown et al., 2014; Deyrup, 2016; Wetterer, 2020). *Odontomachus haematodus* is native to South America, whereas populations from eastern Louisiana to the Florida Panhandle were considered to be exotic (MacGown et al., 2014; Deyrup, 2016). In the case of *Odontomachus bauri* and *O. ruginodis*, both were introduced to the Galapagos Islands from the Neotropical region, and the Florida population of *O. ruginodis* was introduced from the West Indies (Brown Jr, 1976; Herrera, Longino & Dekoninck, 2014; Deyrup, 2016; Wetterer, 2020). *Odontomachus simillimus* is a tramp species distributed throughout the Indo-Pacific, and was introduced to Seychelles (Brown Jr, 1976; Framenau & Thomas, 2008; Fisher & Smith, 2008). *Odontomachus troglodytes* is native to sub-Saharan African, while the Madagascar population was possibly introduced (Fisher & Smith, 2008).Therefore, as this species group appears to be highly invasive, correct identification and a thorough investigation of this new ant in Taiwan is urgently needed. In this study, we aimed to (1) identify the recently introduced *Odontomachus* species in Taiwan, as well as its putative source location; (2) characterize the potential distribution range of this species in Taiwan by using ecological niche modelling; and (3) determine whether a niche shift occurred between its native range and Taiwan. The species was identified by morphological comparison and phylogenetic analyses. Ecological niche modelling was applied to predict the potential distribution of this species in Taiwan and examine the niche shift hypothesis by calculating niche similarity between native and exotic populations. Understanding the potential distribution of the newly colonized ant in Taiwan may lead to a better management strategy for the ants and ultimately minimize its environmental impact.

## Material and Methods

### Collection localities and DNA sequencing

A total of 26 specimens of *Odontomachus* ants were collected from Chiayi (*n* = 13) (23.49101°N, 120.47183°E) and Kaohsiung (*n* = 13) (22.619682°N, 120.377824°E) in southwestern Taiwan. In Kaohsiung, many nests were discovered, and all samples were from the same nest, while in Chiayi, all samples were trailing ants outside of the nest. Whether they belonged to the same nest is uncertain. All the specimens collected were preserved in 95% ethanol and stored at −20 °C. The species was inspected by morphological characters using keys and diagnoses provided in Brown Jr (1976) under a stereo microscope (Leica S9D, Switzerland) and differences were described between the newly exotic ant and other *Odontomachus* species. Then, an identification key was developed to identify Ponerinae trap-jaw ants in Taiwan.

We then sequenced the cytochrome oxidase I gene (*COI*) to confirm the morphological species identification. The genomic DNA was extracted from the right mid-leg of worker ants using the NautiaZ Tissue DNA Mini Kit (Nautia, Taipei, Taiwan) following the procedure of other studies (Chen et al., 2017). Polymerase chain reactions (PCR) were performed to amplify the *CO1* gene following the protocol from Vrijenhoek (1994). The pair of primers, HCO2198 (5′-TAAACTTCAGGGTGACCAAAAAATCA-3′) and LCO1490 (5′-GGTCAACAAATCATAAAGATATTGG-3′), were used for the following experiments. PCR reactions were conducted in 12.5 µl reaction volumes containing 2.5 mM MgCl_2_, 5 pmol of each primer, 20 µM dNTPs, 10mM TrisHCl (pH = 8.3), 50 mM KCl, 2 µl of genomic DNA, and 1 unit of TaqDNA polymerase (Platinum^®^ Taq DNA Polymerase; Invitrogen, Waltham, MA, USA). The PCR condition comprises one cycle for denaturation at 94 °C for 5 min, followed by 35 cycles of 40 s at 94 °C, 40 s at 45 °C, and 60 s at 72 °C, then a final process of 5 min at 72 °C for an extension. The products of PCR were subsequently sequenced using ABI 3730 DNA Sequencer (Tri-I Biotech). The sequences generated were compared with known sequences of the species from NCBI’s GenBank. All the sequences were deposited in GenBank, with accession numbers listed in Table 1.

**Table 1:** Species, accession number, voucher number, collected localities used in this study.

Organism	Sequence_ID	Specimen-voucher	Locality
*Odontomachus monticola*	ON804843	OMC1	23.707938°N, 120.70812°E
*Odontomachus monticola*	ON804844	OMC2	23.707938°N, 120.70812°E
*Odontomachus monticola*	ON804845	OMN1	24.648552°N, 121.432824°E
*Odontomachus monticola*	ON804846	OMN2	24.648552°N, 121.432824°E
*Odontomachus monticola*	ON804847	OMS1	22.41437°N, 120.72939°E
*Odontomachus troglodytes*	ON804848	OTC1	23.49101°N, 120.47183°E
*Odontomachus troglodytes*	ON804849	OTC2	23.49101°N, 120.47183°E
*Odontomachus troglodytes*	ON804850	OTC3	23.49101°N, 120.47183°E
*Odontomachus troglodytes*	ON804851	OTC4	23.49101°N, 120.47183°E
*Odontomachus troglodytes*	ON804852	OTC5	23.49101°N, 120.47183°E
*Odontomachus troglodytes*	ON804853	OTC6	23.49101°N, 120.47183°E
*Odontomachus troglodytes*	ON804854	OTC7	23.49101°N, 120.47183°E
*Odontomachus troglodytes*	ON804855	OTC8	23.49101°N, 120.47183°E
*Odontomachus troglodytes*	ON804856	OTC9	23.49101°N, 120.47183°E
*Odontomachus troglodytes*	ON804857	OTC10	23.49101°N, 120.47183°E
*Odontomachus troglodytes*	ON804858	OTC11	23.49101°N, 120.47183°E
*Odontomachus troglodytes*	ON804859	OTC12	23.49101°N, 120.47183°E
*Odontomachus troglodytes*	ON804860	OTC13	23.49101°N, 120.47183°E
*Odontomachus troglodytes*	ON804861	OTK1	22.619682°N, 120.377824°E
*Odontomachus troglodytes*	ON804862	OTK2	22.619682°N, 120.377824°E
*Odontomachus troglodytes*	ON804863	OTK3	22.619682°N, 120.377824°E
*Odontomachus troglodytes*	ON804864	OTK4	22.619682°N, 120.377824°E
*Odontomachus troglodytes*	ON804865	OTK5	22.619682°N, 120.377824°E
*Odontomachus troglodytes*	ON804866	OTK6	22.619682°N, 120.377824°E
*Odontomachus troglodytes*	ON804867	OTK7	22.619682°N, 120.377824°E
*Odontomachus troglodytes*	ON804868	OTK8	22.619682°N, 120.377824°E
*Odontomachus troglodytes*	ON804869	OTK9	22.619682°N, 120.377824°E
*Odontomachus troglodytes*	ON804870	OTK10	22.619682°N, 120.377824°E
*Odontomachus troglodytes*	ON804871	OTK11	22.619682°N, 120.377824°E
*Odontomachus troglodytes*	ON804872	OTK12	22.619682°N, 120.377824°E
*Odontomachus troglodytes*	ON804873	OTK13	22.619682°N, 120.377824°E

### Identification of putative source population

In order to identify the exotic species in Taiwan, sympatric or nearby species, and species with the same identification character, were included in the analyses. Therefore, the sequences of the *COI* gene of four congeners were selected in the analyses, which included one native species of Taiwan (*O. monticola*), one species distributed close to Taiwan (*O. simillimus* is distributed in Asia), and two species with a similar metasternal process (*O. troglodytes* in Africa and Madagascar, and *O. haematodus* from Neotropical). Putative source population of newly exotic ants can be inferred by comparing sequence similarity among different populations of the same species. The sequences were aligned by CLUSTAL W in MEGA-X (Kumar et al., 2018), and those with redundant or missing regions were trimmed or removed in the following analyses.

To investigate the phylogenetic relationship between different populations of *O. troglodytes* and other *Odontomachus* species, a maximum likelihood tree was built using RAxML with 100,000 bootstrapping replicates as validation (Stamatakis, 2014). Model selection was performed using jModelTest v2.1.10 (Guindon & Gascuel, 2003; Darriba et al., 2012) to find a best-fit model for phylogenetic tree construction based on the Bayesian information criterion (Darriba et al., 2012). Moreover, a Bayesian phylogenetic tree was constructed using MrBayes 3.2.7a (Ronquist & Huelsenbeck, 2003), while posterior probability was estimated by Metropolis-coupled Markov chain Monte Carlo analysis with four chains running for 1,000,000 cycles. The visualization and compilation of both phylogenetic trees were conducted on Interactive Tree Of Life (http://itol.embl.de) (Letunic & Bork, 2019). To further examine the relationship among populations of *O. troglodytes*, haplotype networks of the *COI* gene were constructed using 89 sequences of *O. troglodytes* by R package ‘pegas’ (Paradis, 2010).

### Ecological niche modelling and niche shift analysis

The occurrence data included records from the Global Ant Biodiversity Informatics (GABI) project (105 sites, Guénard et al., 2017), a previous study (174 sites, Bates, Ollier & Bertelsmeier, 2020), and collection sites in Taiwan (five sites). After combining all three datasets and removing duplicated data, 209 occurrence points were obtained (Fig. S1A). To avoid spatial autocorrelation (Boria et al., 2014) while maintaining most of the occurrence data in Taiwan for the following analyses, the initial set of occurrence data was thinned *via* a 2-km distance filter by R package ‘spThin’ (Aiello-Lammens et al., 2015). After compilation and rarefaction, 156 occurrence points were retained for subsequent ecological niche modelling (Fig. S1B).

A total of 20 environmental variables, consisting of nineteen climatic factors and one elevation layer, were downloaded from WorldClim (https://www.worldclim.org/) for niche model construction. Considering the recorded presence of *O. troglodytes* in suburban habitats (Fisher & Smith, 2008) and the strong influence of human land use on ant diversity (Baidya & Bagchi, 2022), the dataset of human modification and settlement on terrestrial lands (Kennedy et al., 2020), which provided a cumulative measure of the impacts of an industrial building, agriculture, transportation and other human settlements, was also included in the following analysis to evaluate the influence of human activity on the distribution of ants. To avoid impacts of multilinearity among 21 variables, variance inflation factors were calculated for rarefaction using the R package ‘usdm’ (Naimi et al., 2014). The calculation excluded highly correlated factors, and ten variables were retained (Table S1). Ecological niche modelling was performed using the maximum entropy approach in MaxEnt, a widely applied software that models potential species distribution using presence-only data (Philips & Dudík, 2008).

The niche model would be validated while the random test percentage was set as 10, which meant the occurrence data was split into ‘training’ and ‘testing’ datasets, with 90% of the occurrence data used for training the model and 10% used to test the model. The jackknife tests evaluated the contribution of each environmental variable. The visualized results of ecological niche modelling were plotted in R (R Core Team, 2021).

A principal component analysis (PCA) was performed based on bioclimatic variables from the ecological niche modelling analysis to examine the environmental variability across the different distribution areas. To further examine whether niche shift exists between native and non-native populations of *O. troglodytes*, niche shift analysis was conducted using modified codes from Bates, Ollier & Bertelsmeier (2020). A between-class analysis was performed to identify the axis separating native and non-native populations (Broennimann et al., 2012), which is subsequently transformed into densities of occurrences using the R package ‘ecospat’ (Di Cola et al., 2017). The intersection of occurrence densities was determined by Schoener’s D (Schoener, 1968; Warren, Glor & Turelli, 2008) to measure niche overlap in the analysis.

## Results

### Species and putative source identification

**Figure 1:**
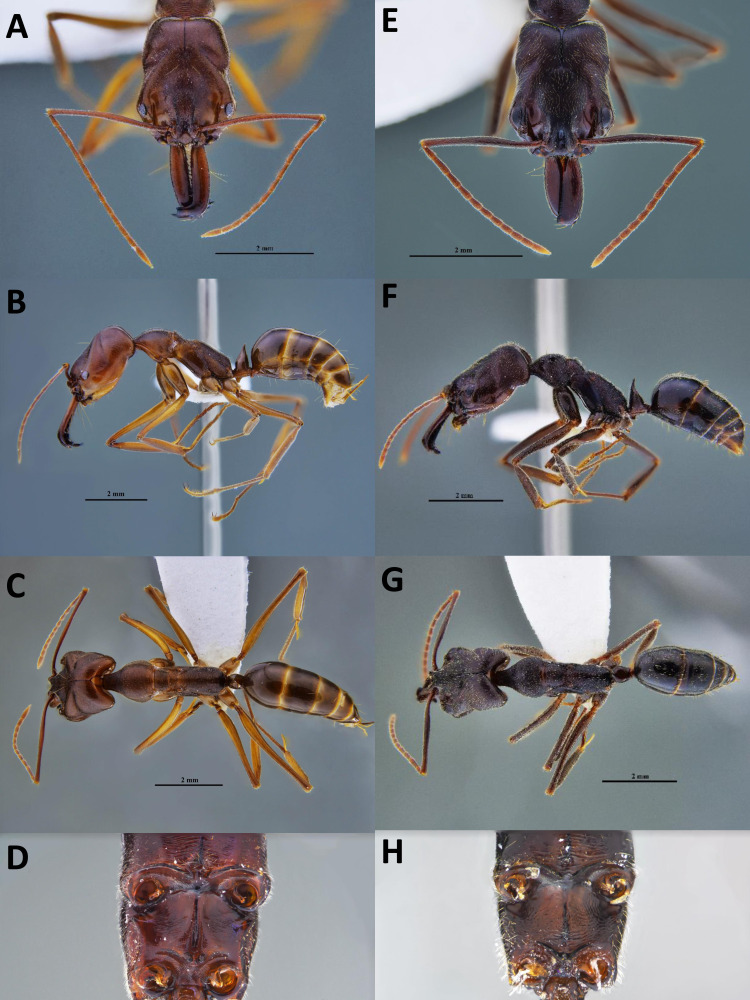
Workers of two *Odontomachus* species in Taiwan. (A–D) *Odontomachus monticola*; (E–H) *Odontomachus troglodytes*. (A, E) Full-face view. (B, F) Lateral view. (C, G) Dorsal view. (D, H) Metasternal process. Photo credit: Fu-ya Chung.

**Figure 2:**
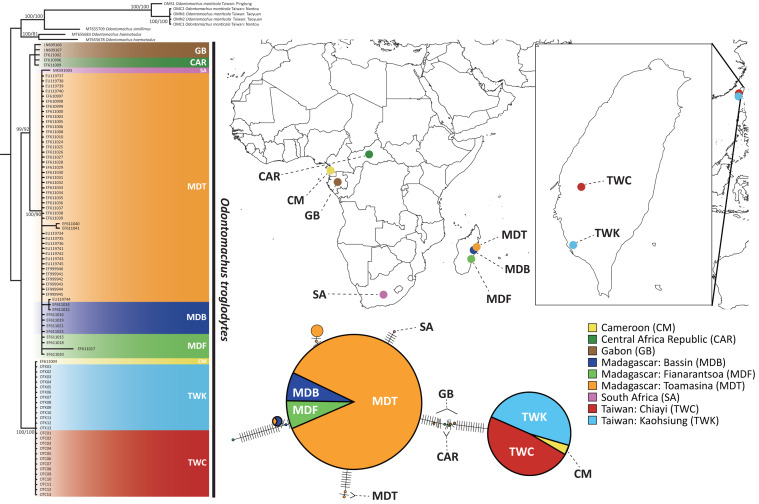
The phylogenetic relationship and haplotype network of *Odontomachus troglodytes*. Populations of *O. troglodytes* in Taiwan form a monophyletic clade with one sample from Cameroon based on the same tree topology of maximum likelihood and Bayesian inference tree using mitochondrial *COI* sequences. The numbers on the nodes represent the branch support value of the likelihood bootstrap/Bayesian posterior probability. In the haplotype network, each bar indicates the corresponding nucleotide change among different haplotypes. Each circle represents a haplotype, whose size is proportional to the numbers of individuals posing this haplotype. The individuals from Taiwan and Cameroon have identical haplotype. The location of each population was marked using different colors.

We first suspected the species as *O. simillimus* by a key to *Odontomachus* of the Indo-Australian region in Brown Jr (1976) due to its distribution in Asia. However, the morphology of the metasternal process (Fig. 1H) showed that the Taiwanese species is more likely to be another closely related species, either *O. haematodus* from Neotropical or *O. troglodytes* from Africa, two species with very similar morphologies. Brown Jr (1976) described their differences: *O. troglodytes* have brown legs, shorter antennal scape, and the sides of a petiolar node are usually more coarsely striated and opaque. However, it was still challenging to identify the species merely by these traits. The comparison of the nucleotide database on GenBank showed that the *COI* sequence of Taiwanese *Odontomachus* is 100% identical corresponding to *O. troglodytes* (voucher CASENT0009457-D01) from Cameroon (Fig. 2), indicating this newly recorded species is *O. troglodytes*. An identification key of Ponerinae trap-jaw ants in Taiwan was constructed, which included three species of *Anochetus* and two species of *Odontomachus* (Documents S1).

The sequence length of *COI* genes was 591 bp after alignment by CLUSTAL W, and a total of 97 sequences were obtained for subsequent analyses. The substitution model selection in jModelTest suggested “HKY + G” as the best model according to the Bayesian information criterion. Both the maximum likelihood tree and Bayesian phylogenetic tree (Fig. 2) showed that the population of Taiwan and one sample from Cameroon are monophyletic with high bootstrap support, separated from all the other samples from Africa. Individuals from Madagascar were clustered with Gabon and Central African Republic of central Africa populations. This finding indicates that central Africa might be the closest origin of this alien species in Taiwan. Furthermore, the haplotype network using *COI* sequences reveals that 26 individuals from Kaohsiung and Chiayi in Taiwan shared an identical haplotype with Cameroon, which is different from the rest of the populations in Africa and Madagascar (Fig. 2).

### Temperature differences and precipitation in summer affect the distribution of *Odontomachus troglodytes*

**Figure 3:**
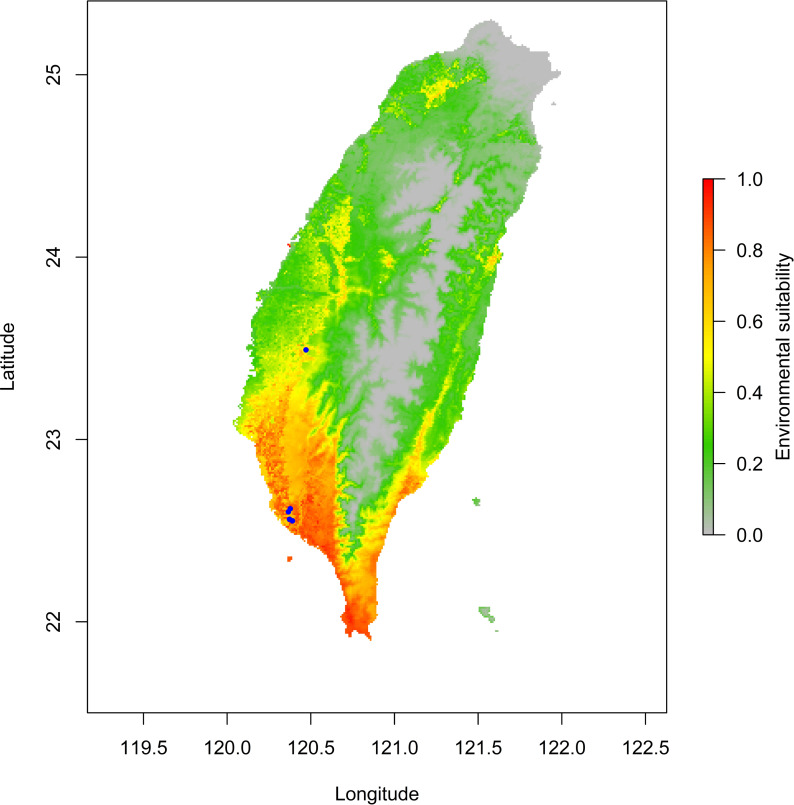
Potential distribution range of *Odontomachus troglodytes* in Taiwan. The blue spots indicate collection sites. The color gradient indicates environmental suitability of *O. troglodytes*, while the areas with high suitability mainly appear in southwestern Taiwan.

The jackknife test results revealed that the analysis’s two variables (BIO18 and BIO03) were the most critical environment predictors (Fig. S2). The environmental variable BIO03 representing isothermality (the ratio of diurnal and annual temperature range), has the most helpful information that is not present in other variables. In contrast, the environmental variable BIO18 (the precipitation of the warmest quarter) has the most useful information by itself. Therefore, isothermality and the precipitation of the warmest quarter contained the most information among selected environmental variables. The relationship of the probability of predicting occurrences for each variable was shown in response curve plots (Fig. S3). For the precipitation of the warmest quarter (BIO18), the logistic outputs increased with the higher variable value until it reached around 1,250 (mm/m^2^), which represents the positive correlation between the predicting probability of occurrence and the value of the variables (Fig. S3A). With a medium level of isothermality, the highest output for isothermality (BIO03) ranges from 60 to 85 (%), indicating a higher prediction probability (Fig. S3B). This finding indicates that the relationships between essential variables and the prediction probability of the MaxEnt model were different. In contrast, human modification and settlement did not contribute much to our modelling. Prediction of possible distribution suggested that the lowland of southern Taiwan are suitable regions for exotic *O. troglodytes* (Fig. 3).

### Niche shift and expansion patterns

**Figure 4:**
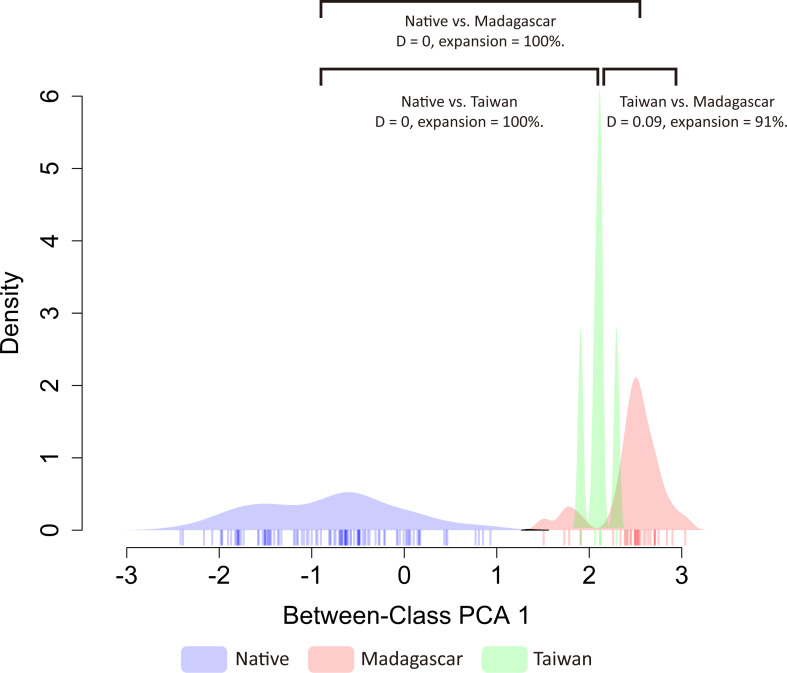
Similar patterns of niche overlap and niche expansion among three populations. The color of each polygon represents the status of each population, while the blue one is the native population, the red and green ones represent the non-native population from Madagascar and Taiwan, separately.

Based on the principal component analysis, both exotic populations (Madagascar and Taiwan) occupy different habitats than the native range, and also inhabit different environments (Fig. S4). The first two axes of the PCA analysis explained 65.6% of the total variation of environmental variability across the distribution ranges of *O. troglodytes* (PC1 = 35.2% and PC2 = 30.4%). The first component (PC1) was primarily explained by the mean diurnal range (BIO02), whereas the second was principally loaded by the max temperature of the warmest month (BIO05) (Fig. S5). To evaluate niche shift patterns of *O. troglodytes* after adding invasive records in Taiwan, we applied methods modified by Bates, Ollier & Bertelsmeier (2020) to estimate niche similarity and expansion patterns. When analyzing Taiwan together with the original dataset from Bates, Ollier & Bertelsmeier (2020), continental Africa (native), Madagascar (invasive), and Taiwan (invasive) populations of *O. troglodytes* all showed small *D* overlap and high niche expansion under PC1 factors (Fig. 4). Under PC2 factors, Taiwan again showed small *D* overlap and high niche expansion compared to continental Africa and Madagascar; however, continental Africa and Madagascar now showed an increase in *D* overlap and no niche expansion (Fig. S6).

## Discussion

### Long-distance invasion and low genetic diversity of *Odontomachus troglodytes*

Using morphological and genetic analyses, we describe *O. troglodytes* as a new alien species in Taiwan. From the phylogenetic and haplotype analyses, the *COI* sequences of 26 individuals from two locations in Taiwan were identical and shared the same haplotype with a sample from Cameroon. For newly exotic species, the founder effect can be observed with low genetic diversity in the exotic populations, and haplotypes of exotic populations might derive from the most common ones of native populations. *Odontomachus troglodytes* are widely distributed in Africa. However, we only have minimal samples from this area and one from Cameroon. In our study, Madagascar origin can be excluded or, more likely, the ant was introduced from Cameroon or nearby, although more sampling from its native range is needed.

In addition to the invasive record in Madagascar, *Odontomachus troglodytes* discovered in Taiwan are the second invasive population outside their native area. This study revealed a long-distance invasive event from West-Central Africa to Taiwan, even though the commercial trade during these years was not frequent between Cameroon and Taiwan (https://cuswebo.trade.gov.tw/FSC3010F/FSC3010F), or the ant has traversed through multiple trade routes. Although no literature has recorded that this ant was found in surrounding area of Taiwan, such as China or Japan, the pet trade in ants is extremely common recently. From 2002 to 2017, at least 520 ant species from 95 genera were sold online, including some *Odontomachus* species (Gippet & Bertelsmeier, 2021). Therefore, it is also possible that this ant may have been illegally transported to Taiwan for the purpose of being kept as a pet (Gippet & Bertelsmeier, 2021). The ants might be imported *via* the Port of Kaohsiung, close to the area occupied by *O. troglodytes.* Outside of Kaohsiung and Chiayi, there is no indication that this species has spread to other areas. According to our findings, humans may have transported the individuals that colonized Chiayi as the two localities shared the same haplotype. The results of this study implicate the importance of correct identification of exotic species, and further can be applied to invasive risk management.

### The potential niche shifts of exotic *Odontomachus troglodytes*

The result shows that there is no niche overlap between populations in native regions and Taiwan, indicating a niche shift has occurred in the newly introduced Taiwanese population (Fig. 4, Fig. S6). On the other hand, the finding also suggests low niche similarity between the two non-native populations, Taiwan and Madagascar, which is consistent with the results of the principal component analysis. Overall, the results of niche shift analyses support that low niche overlap and high niche expansion among native and two exotic populations of *O. troglodytes*. *Odontomachus troglodytes* colonized Madagascar and displays significant niche expansion and no niche overlap between native and non-native populations (Bates, Ollier & Bertelsmeier, 2020). After incorporating the new distribution data in Taiwan from this study, the results remained congruent. A niche shift was also found between Madagascar and Taiwan, and the two populations also occupy different habitats in PCA analysis. Although *O. troglodytes* in Cameroon and Taiwan share identical haplotypes and are closely related populations, after colonizing Taiwan from Cameroon, they quickly adapted to the new environment. The result indicated that *O. troglodytes* might adapt to various habitat types during the invasion. Given its wide native distribution range, this tolerance to a wide variety of habitats may represent a pre-adapted ability to become invasive. Some ant species have experienced niche shift between native and non-native populations, including climate shift (Kumar et al., 2015) or diet shift (Balzani et al., 2021). In niche shift analysis in our study, although the exotic *O. troglodytes* might have an established population in Taiwan, the distribution of ants is still limited due to the recent colonization. The few sample localities compared to the original wide distribution in Africa might cause bias estimation in the niche shift analysis.

### The potential impacts of *Odontomachus troglodytes* on native species and human

*Odontomachus haematodus*, the invasive congener in North America, is an aggressive stinger that could cause pain if the nest is disturbed (MacGown et al., 2014). In Taiwan, *O. troglodytes* show similar aggressive behaviour to disturbance. Because *O. troglodytes* mainly inhabits and nests underground, it could become a harassment to humans, especially if they adapt to urban areas with plantations. Because the newly colonized populations are close to human area, several cases showed that alate queens of *O. troglodytes* can intrude indoors by nuptial flight which might be attracted by light in the house. However, the settlement of the ant in an indoor environment is not confirmed.

*Odontomachus troglodytes* was not on the list of the Global Invasive Species Database developed by IUCN. Therefore, this alien species might be considered harmless to the local ecosystems or native species. However, our field observation in Taiwan found that *O. troglodytes* can capture prey efficiently, and the prey included highly mobile species (*e.g.*, leafhoppers, midges and blow flies) (Fig. S7). Therefore, more studies are needed to evaluate if they pose a significant threat to local invertebrates.

Previous studies showed that *O. troglodyes* is polygynous, their colonies could possess over one thousand workers, and their workers are potentially fertile, producing males (Ledoux, 1952; Colombel, 1970; Colombel, 1972; Schmidt & Shattuck, 2014). These biological characteristics may indicate the potential for population expansion. However, a detailed observation of this species’ life history and ecological traits in Taiwan is still lacking. Based on species distribution modelling, lowland areas of southern Taiwan are highly likely to be occupied by exotic *O. troglodytes*. Therefore, long-term monitoring of population dynamics and observation of life history traits of this species are required for future interspecific competition with native species and conservation risk assessment.

## Conclusions

This study discovered the first record of *Odontomachus troglodytes* in Taiwan based on morphological comparison and phylogenetic analysis. These findings confirm a new alien ant species in Taiwan, its putative source from central Africa, and provide related ecological information. The possible distribution of *O. trolglodytes* is predicted through ecological niche modelling, which mainly appears in southern Taiwan. The niche shift hypothesis was tested by calculating niche similarity between native and exotic populations, and the results indicated a significant shift of ecological niches within this species. Moreover, we suggest monitoring new exotic species is essential for evaluating the potential threat in invasion ecology.

##  Supplemental Information

10.7717/peerj.14718/supp-1 Document S1 Identification key to Ponerinae trap-jaw ants in Taiwan Modified from keys in Chen et al. (2019), Eguchi, Bui & Yamane (2014), Leong et al. (2018) and Schmidt & Shattuck (2014)

10.7717/peerj.14718/supp-2 Figure S1 Global occurrence data of *Odontomachus troglodytes* from multiple data sources (A) The occurrence data were from Global Ant Biodiversity Informatics (GABI) project (marked with purple spots), previous study (orange spots), and collection localities in this sutdy (green spots). The dataset was thinned using a distance-based algorithm, with 204 occurrence points. (B) Compiled occurrence data for subsequent model construction. After rarefaction, the ûnal dataset was composed of 156 occurrences points. The red spots represent the occurrence records classiûed as exotic species by Antmaps ( https://antmaps.org/), while the blue spots are native occurrence data.

10.7717/peerj.14718/supp-3 Figure S2 The ratio of diurnal and annual temperature range as well as the precipitation of warmest quarter contain most information among selected environmental variables for the ecological niche model of *Odontomachus troglodytes* The aqua, blue, and red bar represent the results without certain variable, with certain variable, and with all variables, respectively.

10.7717/peerj.14718/supp-4 Figure S3 Response curves reflect how prediction probability affected by both crucial variables to *Odontomachus troglodytes* (A) For BIO18, the predicting probability of occurrence was positively related to the value of the variables; (B) For BIO03, the response curve showed that the peak of the logistic outputs at the middle value of BIO03, indicating a higher predicting probability with a medium level of isothermality.

10.7717/peerj.14718/supp-5 Figure S4 Principal component analysis with nine bioclimatic variables used in ecological niche modelling of *Odontomachus troglodytes* For each dot, the coloration indicates the geographical region where the populations were distributed, while the shapes represented whether the populations were native. The result shows the different environmental preferences between the exotic and native populations of *O. troglodytes*.

10.7717/peerj.14718/supp-6 Figure S5 Contribution of each environmental variable in the principal component analysis

10.7717/peerj.14718/supp-7 Figure S6 Similar patterns of niche overlap and niche expansion among three populations under PC2 factors The color of each polygon represents the status of each population, while the blue one is the native population, the red and green ones represent the non-native population from Madagascar and Taiwan, separately.

10.7717/peerj.14718/supp-8 Figure S7 Prey species of*Odontomachus troglodytes* observed in Taiwan *Odontomachus troglodytes* may prey on live or dead prey, and more studies are needed. Prey species were observed: (A) leafhopper (Hemiptera: Cicadellidae); (B) midge (Diptera: Chironomidae); (C) blow fly (Diptera: Calliphoridae). Photo credit: Kai-Wei Chan.

10.7717/peerj.14718/supp-9 Table S1 Ten environmental variables for ecological niche modelling analysis Twenty-one environmental variables were downloaded from WorldClim, then these variables were fltered using R to detect the multicollinearity. Totally ten variables were selected for the following niche modelling analysis.

10.7717/peerj.14718/supp-10 Data S1 The raw data used in ecological niche modelling The occurrence data was thinned via a 2-km distance.

10.7717/peerj.14718/supp-11 Data S2 The *COI* genes of *Odontomachus* species in Taiwan The sequences were uploaded on GenBank.

## Additional Information and Declarations

### DNA Deposition

The following information was supplied regarding the deposition of DNA sequences: The sequences described here are available *via* GenBank: ON804843–ON804873.

### Data Availability

The following information was supplied regarding data availability: Raw data is available in the Supplemental Files.
