# Supplementary material for: Putative source and niche shift pattern of a new alien ant species (Odontomachus troglodytes) in Taiwan
Source: PeerJ. 2023 Feb 6;11:e14718. doi: 10.7717/peerj.14718 (PMC9910184; doi:10.7717/peerj.14718)
Supplement: Document S1 — Modified from keys in Chen et al. (2019), Eguchi, Bui & Yamane (2014), Leong et al. (2018) and Schmidt & Shattuck (2014) [file peerj-11-14718-s001.docx]

Identification key to Ponerinae trap-jaw ants in Taiwan

(Modified from keys in Chen et al. (2019), Eguchi et al. (2014), Leong et al. (2018), and Schmidt & Shattuck (2014))

1. In full-face view, vertex without a median longitudinal carina; the posterior surface of the head lacking a pair of dark apophyseal lines...……………2 (Genus *Anochetus*)

- In full-face view, vertex with a median longitudinal carina which meet preoccipital carina; the posterior surface of the head with a pair of dark converging apophyseal lines………………….…….…….…………………………4 (Genus *Odontomachus*)

2. Apical portion of mandible with two distinct teeth and a very small intercalary tooth; antennal scape long, exceeding posterior lobe of head by more than twice length of 2nd antennal segment; petiolar node conical shape and sharp……………..…………………………………….……..……*A. risii* Forel, 1900

-Apical portion of mandible with three strong teeth; antennal scape short, not reaching posterior lobe; petiole node without conical shape in frontal view……..…..….….….3

3. Propodeal corner with a pair of spines; eye small, the maximum diameter of eye narrower than the width of scape at it midlength...…………*A. subcoecus* Forel, 1912

- Propodeal corner without spine; eye large, the maximum diameter of eye broader than the width of scape at it midlength………………………………………….*A. lanyuensis* Leong et al., 2018

4. Subapical tooth of mandible prominent and truncated, masticatory margin with 8-12 distinct denticles, pronotum transversely striated (Fig. 2A-C)………..…………………………………………………*O. monticola* Emery, 1892

- Subapical tooth of mandible blunt and indistinct, denticles on masticatory margin reduced, pronotum longitudinally striated (Fig. 2E-G)…………………………………………………..........*O. troglodytes* Santsch, 1914
