# Supplementary figures and images for: Putative source and niche shift pattern of a new alien ant species (Odontomachus troglodytes) in Taiwan
Source: PeerJ. 2023 Feb 6;11:e14718. doi: 10.7717/peerj.14718 (PMC9910184; doi:10.7717/peerj.14718)

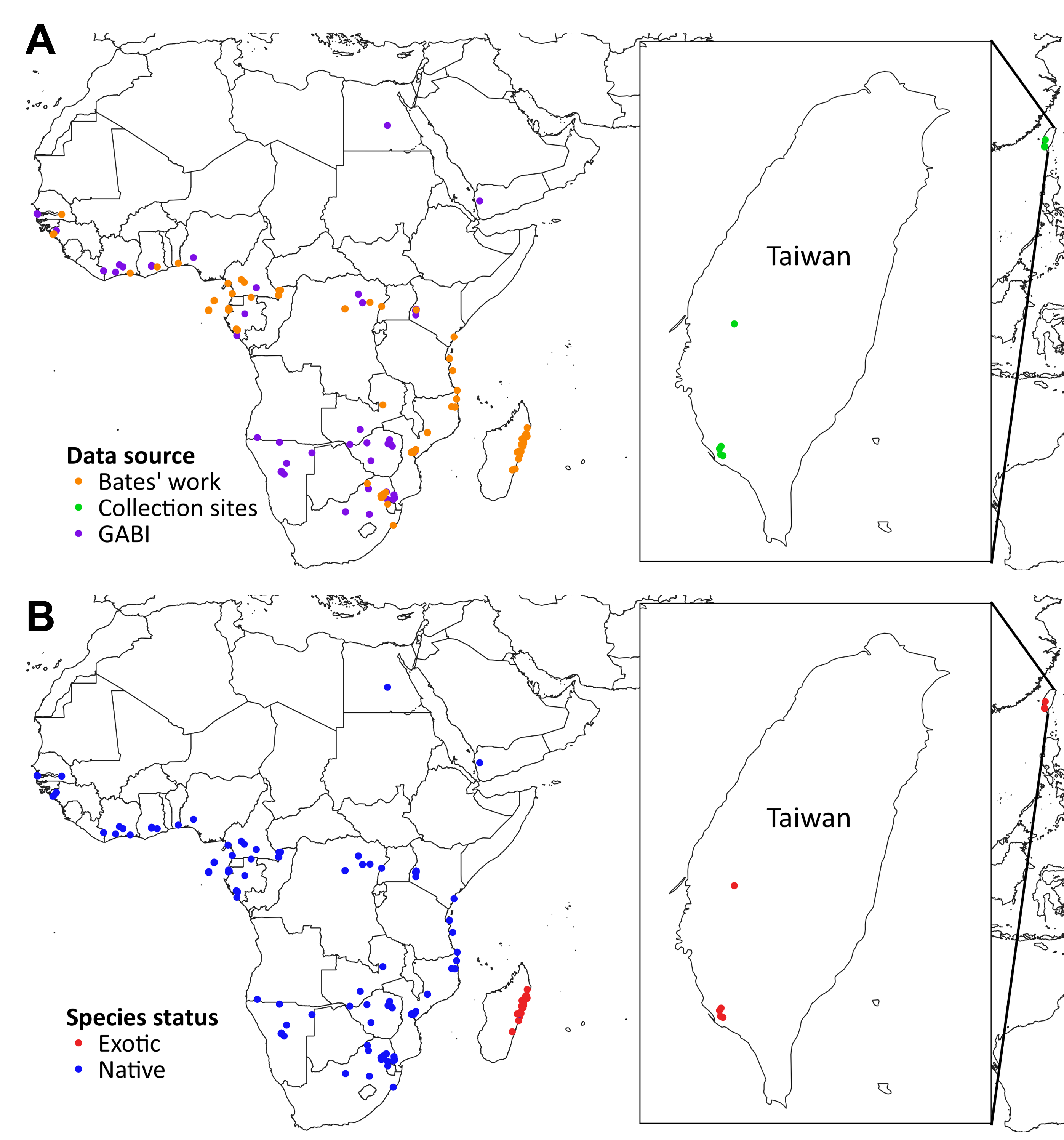

Supplement: Figure S1 — (A) The occurrence data were from Global Ant Biodiversity Informatics (GABI) project (marked with purple spots), previous study (orange spots), and collection localities in this sutdy (green spots). The dataset was thinned using a distance-based algorithm, with 204 occurrence points. (B) Compiled occurrence data for subsequent model construction. After rarefaction, the ûnal dataset was composed of 156 occurrences points. The red spots represent the occurrence records classiûed as exotic species by Antmaps ( https://antmaps.org/), while the blue spots are native occurrence data. [file peerj-11-14718-s002.tif]

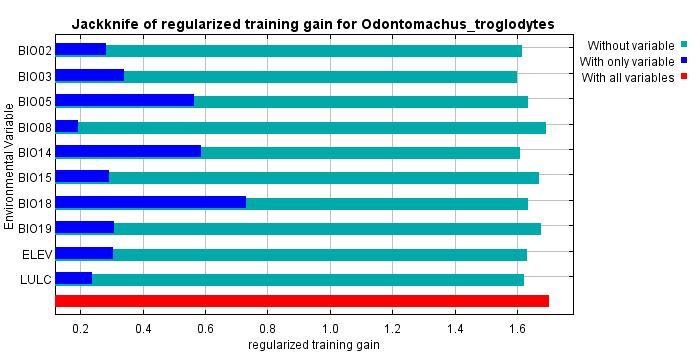

Supplement: Figure S2 — The aqua, blue, and red bar represent the results without certain variable, with certain variable, and with all variables, respectively. [file peerj-11-14718-s003.png]

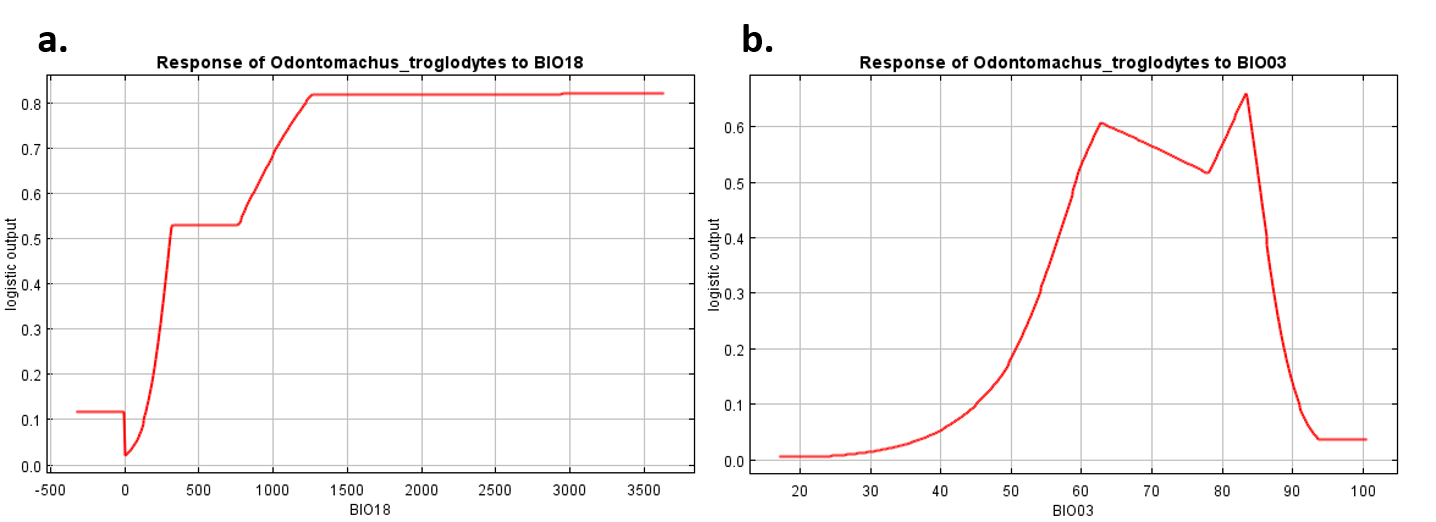

Supplement: Figure S3 — (A) For BIO18, the predicting probability of occurrence was positively related to the value of the variables; (B) For BIO03, the response curve showed that the peak of the logistic outputs at the middle value of BIO03, indicating a higher predicting probability with a medium level of isothermality. [file peerj-11-14718-s004.png]

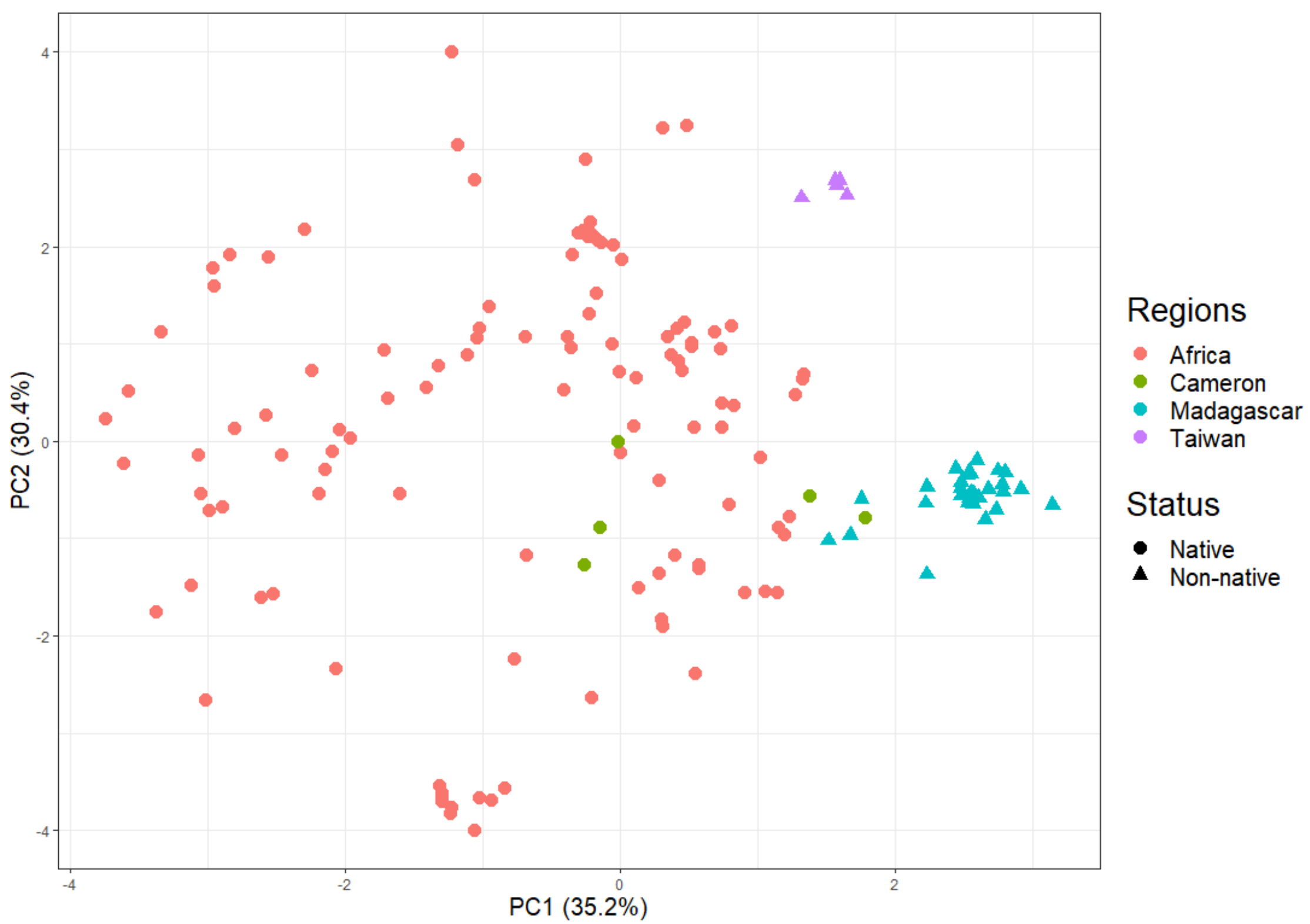

Supplement: Figure S4 — For each dot, the coloration indicates the geographical region where the populations were distributed, while the shapes represented whether the populations were native. The result shows the different environmental preferences between the exotic and native populations of O. troglodytes. [file peerj-11-14718-s005.pdf]

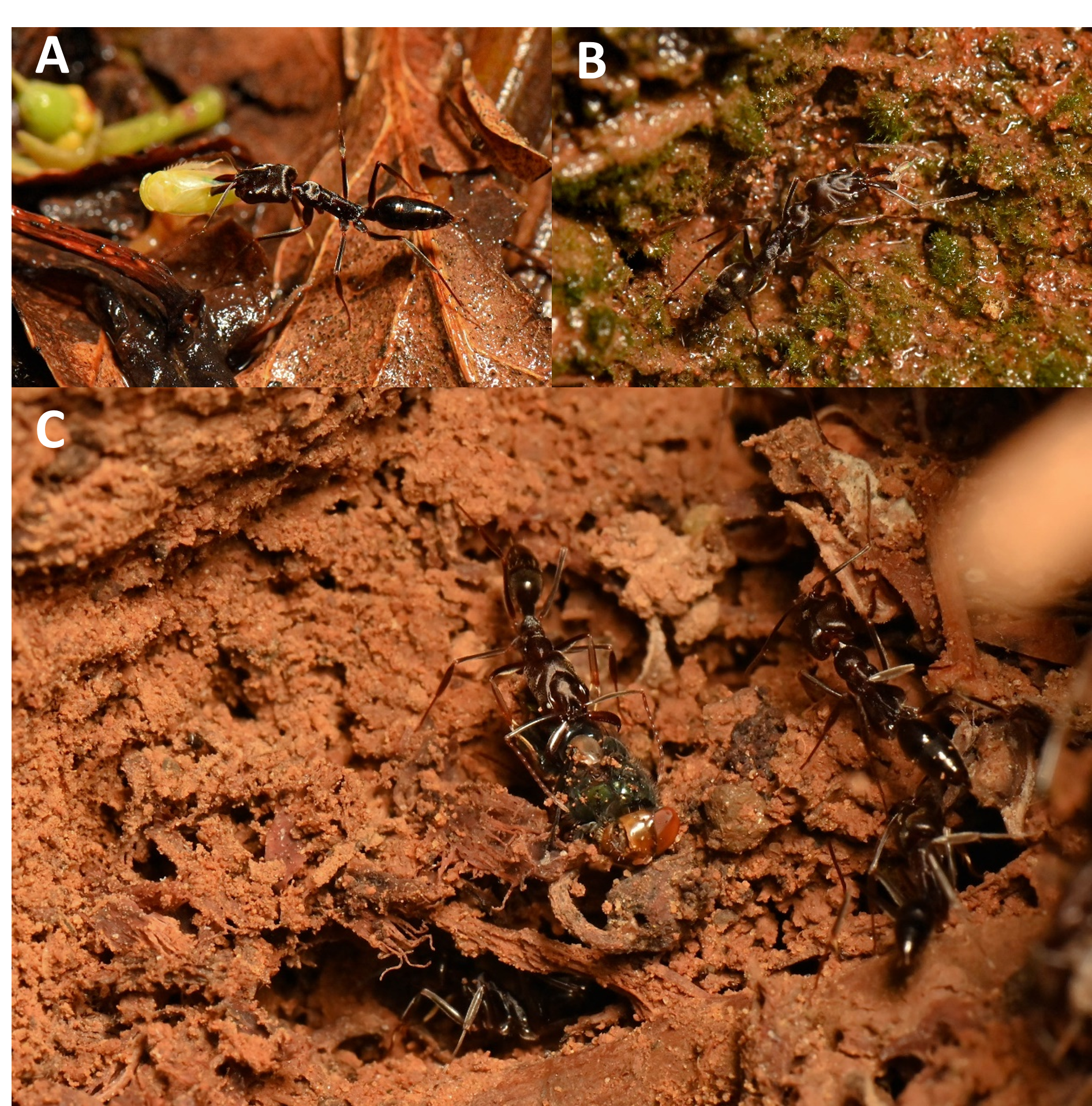

Supplement: Figure S7 — Odontomachus troglodytes may prey on live or dead prey, and more studies are needed. Prey species were observed: (A) leafhopper (Hemiptera: Cicadellidae); (B) midge (Diptera: Chironomidae); (C) blow fly (Diptera: Calliphoridae). Photo credit: Kai-Wei Chan. [file peerj-11-14718-s008.tif]
