# Supplementary material for: Putative source and niche shift pattern of a new alien ant species (Odontomachus troglodytes) in Taiwan
Source: PeerJ. 2023 Feb 6;11:e14718. doi: 10.7717/peerj.14718 (PMC9910184; doi:10.7717/peerj.14718)
Supplement: Figure S6 — The color of each polygon represents the status of each population, while the blue one is the native population, the red and green ones represent the non-native population from Madagascar and Taiwan, separately. [file peerj-11-14718-s007.pdf]

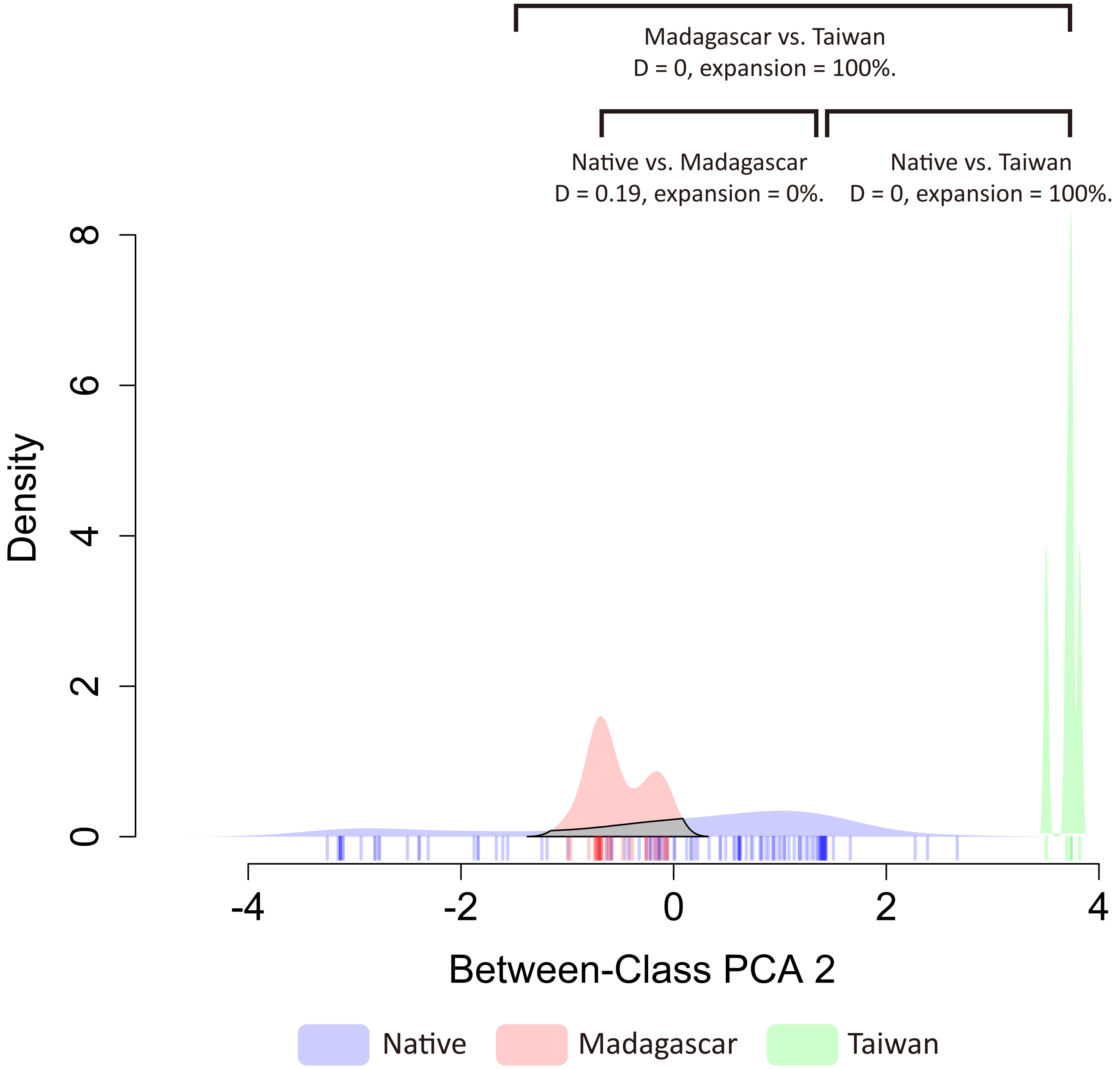

Madagascar vs. Taiwan
D = 0, expansion = 100%.
Native vs. Madagascar
D = 0.19, expansion = 0%.
Native vs. Taiwan
D = 0, expansion = 100%.
Density
0
2
4
6
8
-4
-2
0
2
4
Between-Class PCA 2
Native
Madagascar
Taiwan
