# Supplementary material for: Putative source and niche shift pattern of a new alien ant species (Odontomachus troglodytes) in Taiwan
Source: PeerJ. 2023 Feb 6;11:e14718. doi: 10.7717/peerj.14718 (PMC9910184; doi:10.7717/peerj.14718)
Supplement: Table S1 — Twenty-one environmental variables were downloaded from WorldClim, then these variables were fltered using R to detect the multicollinearity. Totally ten variables were selected for the following niche modelling analysis. [file peerj-11-14718-s009.docx]

| Variable code | Environmental variable |
| --- | --- |
| BIO02 | Mean Diurnal Range (Mean of monthly (max temp – min temp)) |
| BIO03 | Isothermality (Mean Diurnal Range/ Temperature Annual Range) (*100) |
| BIO05 | Max Temperature of Warmest Month |
| BIO08 | Mean Temperature of Wettest Quarter |
| BIO14 | Precipitation of Driest Month |
| BIO15 | Precipitation Seasonality (Coefficient of Variation) |
| BIO18 | Precipitation of Warmest Quarter |
| BIO19 | Precipitation of Coldest Quarter |
| ELEV | Elevation |
| LULC | Land use and Land cover |
